# Impact of tricuspid regurgitation on late right ventricular failure in left ventricular assist device patients ~can prophylactic tricuspid annuloplasty prevent late right ventricular failure? ~

**DOI:** 10.1186/s13019-021-01492-0

**Published:** 2021-04-20

**Authors:** Taro Nakazato, Daisuke Yoshioka, Koichi Toda, Shigeru Miyagawa, Satoshi Kainuma, Takuji Kawamura, Ai Kawamura, Noriyuki Kashiyama, Takayoshi Ueno, Toru Kuratani, Yasushi Sakata, Yoshiki Sawa

**Affiliations:** 1 Department of Cardiovascular Surgery, Osaka University Graduate School of Medicine, 2-15 Yamadaoka, Suita, 565-0871 Japan; 2 Department of Cardiology, Osaka University Graduate School of Medicine, Suita, Japan

**Keywords:** Left ventricular assist device, Continuous flow, Tricuspid regurgitation, Tricuspid annuloplasty, Right heart failure

## Abstract

**Background:**

In this study, we evaluated the prevalence of tricuspid regurgitation (TR) worsening in patients with left ventricular assist devices (LVADs) and its impact on late right ventricular (RV) failure.

**Methods:**

We enrolled 147 patients of the 184 patients who underwent continuous-flow LVAD implantations from 2005 to March 2018. The prevalence of postoperative TR worsening and late RV failure were retrospectively evaluated.

**Results:**

Concomitant tricuspid annuloplasty (TAP) was performed in 28 of 41 patients (68%) with preoperative TR greater than or equal to moderate (TR group) and in 23 of 106 patients (22%) with preoperative TR less than or equal to mild (non-TR group). Regarding the TR-free rates, despite receiving or not receiving concomitant TAP, there was no significant difference between the 2 groups (TR group: *p* = 0.37; non-TR group: *p* = 0.42). Of the 9 patients with postoperative TR greater than or equal to moderate, late RV failure developed in 3 patients, with TR worsening after RV failure in each case. During follow-up, 16 patients (11%) had late RV failure. As for the late RV failure-free rates, despite receiving or not receiving concomitant TAP, there was no significant difference between the 2 groups (TR group: *p* = 0.37; non-TR group: *p* = 0.96).

**Conclusions:**

TR prognosis was preferable regardless of a patient receiving concomitant TAP; however, the presence of postoperative TR seemed to unrelated to late RV failure. Prophylactic TAP might not be necessary to prevent late RV failure.

**Supplementary Information:**

The online version contains supplementary material available at 10.1186/s13019-021-01492-0.

## Background

Use of left ventricular assist devices (LVADs) has become a standard of care among patients with end-stage heart failure [1, 2]. However, right ventricular (RV) failure after LVAD implantation is an unresolved issue that is associated with significant perioperative mortality and morbidity [3–8]. Approximately 20–30% of patients develop some form of RV failure after LVAD implantation [3, 4]. The right atrium pressure (RAP)/pulmonary capillary wedge pressure (PCWP) ratio, pulmonary artery pulsatility index (PAPi), and right ventricular stroke work index (RVSWI) have been reported as risk factors of postoperative RV failure in patients with LVAD implantation [3, 9, 10]. Late RV failure, which has been recently described, is a refractory RV failure after LVAD implantation, and various factors, including tricuspid diameter, have been reported as predictors [11–14]. It is well known that tricuspid annuloplasty (TAP) for tricuspid regurgitation (TR) at LVAD implantation prevents postoperative acute phase RV failure [15]. However, the prevalence of late TR after TAP and the impact of TAP for preventing late RV failure are still unknown. Therefore, this study aimed to evaluate the prevalence of late TR and its impact on late RV function in patients with LVADs.

## Materials and methods

### Patients

**Fig. 1:**
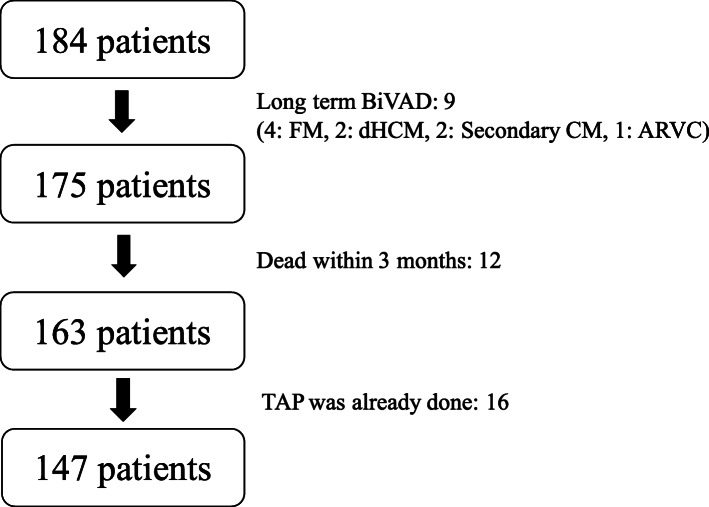
Patients criteria is shown. (Abbreviations, ARVC: arrhythmogenic right ventricular cardiomyopathy, BiVAD: biventricular assist device, CM: cardiomyopathy, dHCM: dilated phase hypertrophic cardiomyopathy, FM: fulminant myocarditis, TAP: tricuspid annuloplasty)

From 2005 to March 2018, excluding patients aged < 10 years, 184 patients underwent continuous-flow LVAD implantation, primarily as bridge-to-transplantation therapy for severe heart failure, at Osaka University Hospital (Fig. 1). Finally, 147 patients (follow-up period: 318 patient-years) were enrolled in this study and the prevalence of tricuspid regurgitation (TR) worsening and its impact on late RV function were evaluated.

### Echocardiographic and catheter parameters

The echocardiographic parameters obtained just before the LVAD implantation were adopted. Postoperative echocardiography was routinely performed at 1 week and 1 month during hospitalization. Additional echocardiography examinations were performed when deemed necessary by the physician. At the outpatient clinic, routine echocardiography was performed approximately every 6 months until heart transplantation. Regarding the etiology of TR, pulmonary hypertension was defined by a mean pulmonary artery pressure ≥ 25 mmHg at rest, measured during right heart catheterization (RHC) [16]. Severe annular dilation was defined as the diameter of annulus at the diastolic phase over 40 mm measured in the 4-chamber view on the echocardiography [17]. As for TR induced by pacing lead, it was based on the information of operation records. The RV end-diastolic dimension (RVDd) was measured in the 4-chamber view. The presence of postoperative TR was defined as a TR severity greater than or equal to moderate in any postoperative phase.

The Right heart catheterization (RHC), which was routinely performed before heart transplantation list registration or LVAD implantation, was adopted. In patients with critical cardiogenic shock, the RHC data before deterioration were used. The parameters of pulmonary artery pressure and PCWP were obtained. The RAP/PCWP ratio, PAPi, and RVSWI were calculated according to previous reports [3, 9, 10].

### Surgical procedures

LVAD implantation was performed via median sternotomy with cardiopulmonary bypass (CPB). In all patients, TAP was performed by ring-annuloplasty. The decision for concomitant TAP was made based on the following criteria. First, TAP was performed in patients whose preoperative TR grade was severe (including severe in the past). Second, regarding moderate TR, we also performed TAP, but the patients in which the reduction of volume overload improved the grade of TR just before surgery were excluded. Concomitant TAP was divided into 2 types: planned TAP which was preoperatively planned; and unexpected TAP, which was not planned preoperatively but was performed for RV failure due to intraoperative TR after the initial attempt to wean from CPB.

### Postoperative management

The definition of late RV failure was inotrope support for more than 3 months after LVAD implantation or a right heart failure that required readmission. Heart failure that required inotropes for left heart failure with elevated PCWP or pulmonary congestion caused by insufficient LVAD pump speed or aortic valve insufficiency was not considered RV failure.

### Statistical analysis

Continuous variables are expressed as mean ± standard deviation and were compared using the Student’s *t*-test for unpaired data where appropriate. Categorical variables were compared using Fisher’s exact test. Kaplan-Meier analysis was used to estimate the overall TR-free and late RV failure-free rate. The TR-free and late RV failure-free rates were compared between the 2 groups using log-rank analysis. Values of *p* < 0.05 were considered significant. The risk factors for late RV failure was analyzed by Cox proportional hazards analysis. The statistical analysis was performed using SAS software (version 14.0.0; SAS Institute, Inc., Cary, NC, USA).

## Results

The preoperative characteristics of patients are shown in Table 1. Forty-one patients had a preoperative TR severity greater than or equal to moderate (TR group), whereas 106 patients had TR severity less than or equal to mild (non-TR group). There was no statistical difference in the mean age, gender, or the etiology of cardiomyopathy between the 2 groups. On preoperative echocardiography, the RVDd and TR pressure gradient (TRPG) were significantly larger in the TR group, although the tricuspid annular plane systolic excursion (TAPSE) score was similar for both groups. For patients with RHC, pulmonary artery pressure was significantly higher in the TR group. Additionally, PCWP and RAP were significantly higher in the TR group. There was no significant difference between 2 groups in terms of the RAP/PCWP ratio, PAPi, and RVSWI. As for laboratory testing, blood urea nitrogen (BUN), serum creatinine (Cr), and brain natriuretic peptide levels were significantly higher in the TR group.

**Table 1:** Patient’s characteristics (pre-LVAD)

	Overall	TR ≥ moderate (*n =* 41)	TR ≤ mild (*n =* 106)	*p* value
Age	41.9 ± 14.3	43.7 ± 13.2	41.2 ± 14.7	0.33
Male	101 (69%)	30 (73%)	71 (67%)	0.55
Etiology				
DCM, n(%)	89 (61%)	26 (63%)	63 (59%)	0.71
dHCM, n(%)	22 (15%)	5 (12%)	17 (16%)	0.80
ICM, n(%)	15 (10%)	2 (5%)	13 (12%)	0.24
Others, n(%)	21 (14%)	8 (20%)	13 (12%)	0.30
Etiology of TR				
pulmonary hypertension	84 (58%)	29 (71%)	55 (52%)	0.04*
severe annular dilation	54 (37%)	29 (71%)	25 (24%)	< 0.0001*
induced by pacing lead	4 (3%)	2 (5%)	2 (2%)	0.31
INTERMACS profile				
profile 1, n(%)	33 (22%)	11 (27%)	22 (21%)	0.51
profile 2, n(%)	51 (35%)	17 (41%)	34 (32%)	0.34
profile 3, n(%)	58 (39%)	10 (24%)	48 (45%)	0.02*
profile 4, n(%)	5 (3%)	3 (7%)	2 (2%)	0.13
IABP, n(%)	32 (22%)	15 (37%)	17 (16%)	0.01*
ECMO, n(%)	11 (7%)	4 (10%)	7 (7%)	0.50
ventilation, n(%)	25 (17%)	10 (24%)	15 (14%)	0.15
pacing device, n(%)	80 (54%)	20 (49%)	60 (57%)	0.46
LVDd (mm)	69.6 ± 12.7	68.1 ± 12.0	70.3 ± 12.9	0.35
LVDs (mm)	64.1 ± 13.6	62.4 ± 13.0	64.8 ± 13.9	0.35
LVEF(%)	20.7 ± 8.6	20.7 ± 9.7	20.7 ± 8.2	0.99
RVDd (mm)	40.0 ± 9.9	45.8 ± 9.8	37.8 ± 9.1	0.0002*
TAPSE (mm/s)	14.3 ± 4.7	14.4 ± 3.9	14.2 ± 4.9	0.89
TRPG (mmHg)	31.4 ± 14.0	38.0 ± 17.3	29.0 ± 11.7	0.0012*
PAP systolic (mmHg)	40.0 ± 13.5	45.3 ± 14.2	37.9 ± 12.8	0.0041*
PAP diastolic (mmHg)	19.7 ± 7.9	22.3 ± 7.8	18.7 ± 7.8	0.02*
PAP mean (mmHg)	27.8 ± 9.8	31.6 ± 9.8	26.3 ± 9.4	0.0045*
PCWP (mmHg)	20.0 ± 8.5	22.6 ± 8.6	19.0 ± 8.3	0.03*
RAP (mmHg)	7.8 ± 4.9	9.2 ± 4.6	7.3 ± 4.9	0.04*
CI(L/min/m2)	2.25 ± 0.77	2.23 ± 0.97	2.26 ± 0.69	0.80
RAP/PCWP	0.40 ± 0.29	0.42 ± 0.24	0.40 ± 0.31	0.65
PAPi	3.80 ± 3.20	3.26 ± 2.80	4.01 ± 3.33	0.23
RVSWI (mmHg×ml/m2)	537 ± 279	567 ± 359	525 ± 242	0.44
BUN (mg/dl)	20.2 ± 12.4	24.4 ± 17.5	18.5 ± 9.3	0.0094*
Cr (mg/dl)	1.06 ± 0.49	1.24 ± 0.67	0.99 ± 0.38	0.0054*
Ccr (ml/min/m2)	71.2 ± 34.5	63.8 ± 34.1	73.9 ± 34.4	0.12
T-Bil (mg/dl)	1.06 ± 0.75	1.25 ± 0.72	0.99 ± 0.75	0.07
BNP (pg/μl)	412 (30.8–4206)	591 (44–3022)	341 (30.8–4206)	0.0006*

*BNP* brain natriuretic peptide, *BUN* blood urea nitrogen, *Ccr* creatinine clearance, *CI* cardiac index, *Cr* serum creatinine, *DCM* dilated cardiomyopathy, *dHCM* dilated phase hypertrophic cardiomyopathy, *ECMO* extracorporeal membrane oxygenation, *IABP* intra-aortic balloon pumping, *ICM* ischemic cardiomyopathy, *INTERMACS* Interagency Registry for Mechanically Assisted Circulatory Support, *LVAD* left ventricular assist device, *LVDd* left ventricular end-diastolic dimension, *LVDs* left ventricular end-systolic dimension, *LVEF* left ventricular ejection fraction, *PAP* pulmonary artery pressure, *PAPi* pulmonary artery pulsatility index, *PCWP* pulmonary capillary wedge pressure, *RAP* right atrial pressure, *RVDd* right ventricular end diastolic dimension, *RVSWI* right ventricular stroke work index, *TAPSE* tricuspid annular plane systolic excursion, *T-Bil* total bilirubin, *TR* tricuspid regurgitation, *TRPG* tricusid regurgitation peak gradient

**Fig. 2:**
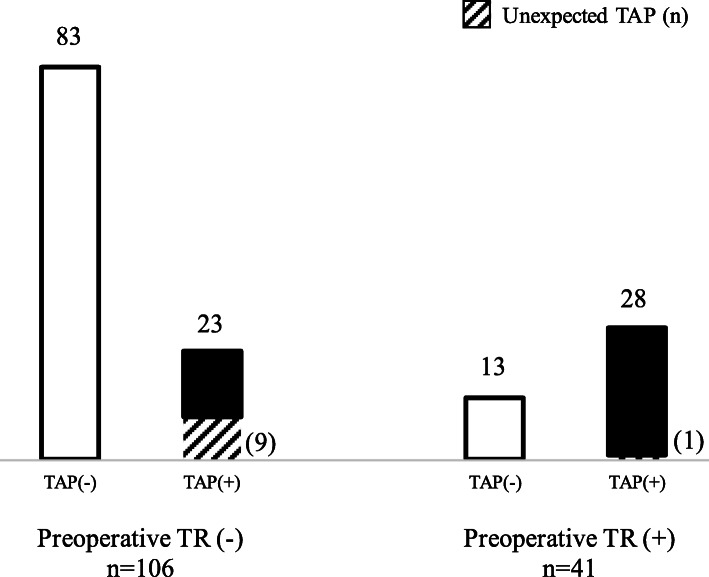
Details on preoperative tricuspid regurgitation (TR) and concomitant TAP of enrolled patients. Preoperative TR (−): TR ≤ mild, preoperative TR (+): TR ≥ moderate. (Abbreviations, TAP: tricuspid annuloplasty)

The prevalence of concomitant TAP is shown in Fig. 2. Of the 147 total patients, 51 (35%) underwent concomitant TAP. Of the 41 patients in the preoperative TR group, 28 (68%) underwent TAP. On the other hand, in the 106 non-TR group patients, TAP was performed in 23 (22%) patients. In the non-TR group, 92 patients were initially scheduled to undergo LVAD implantation without concomitant TAP, whereas 83 (90%) patients successfully underwent LVAD implantation without concomitant TAP. The preoperative characteristics of the 92 patients who were not scheduled for TAP in the non-TR group, based on unexpected TAP status, are shown in Table 2. In patients with unexpected TAP, RVDd was significantly larger and BUN level was significantly higher than in the patients without unexpected TAP (RVDd: 44.4 ± 6.6 vs. 36.8 ± 9.3 mmHg, *p* = 0.04; BUN: 26.8 ± 20.3 vs. 17.7 ± 7.4 mmHg, *p* = 0.0063).

**Table 2:** Comparison between patients of the non-TR group

	without TAP (*n* = 83)	unexpected TAP (*n* = 9)	*p* value
Etiology			
DCM, n(%)	53 (64%)	5 (56%)	0.72
dHCM, n(%)	10 (12%)	2 (22%)	0.33
ICM, n(%)	13 (16%)	0 (0%)	0.35
pacing device, n(%)	45 (54%)	7 (78%)	0.29
TR worsening during LVAD			
(except for TR worsening after RVF)	4 (5%)	0 (0%)	1.00
Pre-LVAD UCG			
LVDd (mm)	69.9 ± 12.9	70.3 ± 11.5	0.92
LVDs (mm)	64.4 ± 14.0	64.8 ± 13.1	0.93
LVEF (%)	21.2 ± 8.7	18.9 ± 4.9	0.44
RVDd (mm)	36.8 ± 9.3	44.4 ± 6.6	0.04*
TAPSE (mm/s)	14.5 ± 5.0	12.0 ± 4.3	0.22
TRPG (mmHg)	29.2 ± 12.0	24.2 ± 9.6	0.24
Pre-LVAD RHC			
PAP systolic (mmHg)	37.9 ± 12.9	31.5 ± 11.5	0.18
PAP diastolic (mmHg)	18.5 ± 7.8	14.4 ± 7.5	0.15
PAP mean (mmHg)	26.2 ± 9.6	21.6 ± 8.5	0.19
PCWP (mmHg)	18.8 ± 7.9	14.6 ± 10.1	0.17
RAP (mmHg)	6.9 ± 4.4	3.9 ± 2.0	0.06
CI(L/min/m2)	2.34 ± 0.72	2.03 ± 0.61	0.25
RAP/PCWP	0.39 ± 0.34	0.33 ± 0.24	0.67
PAPi	4.14 ± 3.33	6.16 ± 4.43	0.12
RVSWI (mmHg×ml/m2)	554 ± 250	458 ± 241	0.30
Pre-LVAD laboratory data			
BUN (mg/dl)	17.7 ± 7.4	26.8 ± 20.3	0.0063*
Cr (mg/dl)	0.98 ± 0.40	1.18 ± 0.30	0.14
Ccr (ml/min/m2)	75.9 ± 35.7	57.4 ± 25.9	0.14
T-Bil (mg/dl)	0.90 ± 0.52	0.84 ± 0.39	0.80
BNP (pg/μl)	449 ± 372	489 ± 425	0.76

*BNP* brain natriuretic peptide, *BUN* blood urea nitrogen, *Ccr* creatinine clearance, *CI* cardiac index, *Cr* serum creatinine, *DCM* dilated cardiomyopathy, *dHCM* dilated phase hypertrophic cardiomyopathy, *ICM* ischemic cardiomyopathy, *LVAD* left ventricular assist device, *LVDd* left ventricular end-diastolic dimension, *LVDs* left ventricular end-systolic dimension, *LVEF* left ventricular ejection fraction, *PAP* pulmonary artery pressure, *PAPi* pulmonary artery pulsatility index, *PCWP* pulmonary capillary wedge pressure, *RAP* right atrial pressure, *RHC* right heart catheterization, *RVDd* right ventricular end diastolic dimension, *RVF* right ventricular failure, *RVSWI* right ventricular stroke work index, *TAPSE* tricuspid annular plane systolic excursion, *T-Bil* total bilirubin, *TR* tricuspid regurgitation, *TRPG* tricusid regurgitation peak gradient, *UCG* ultrasonic echocardiography

The details of patients who had postoperative TR are shown in Supplement 1. Nine patients had a postoperative TR degree greater than or equal to moderate. Two patients in the TR group had postoperative TR, although concomitant TAP and late RV failure developed. On the other hand, one patient in the non-TR group had postoperative TR and late RV failure. However, in all of these patients, the occurrence of TR was recognized after or at the time of RV failure occurrence.

**Fig. 3:**
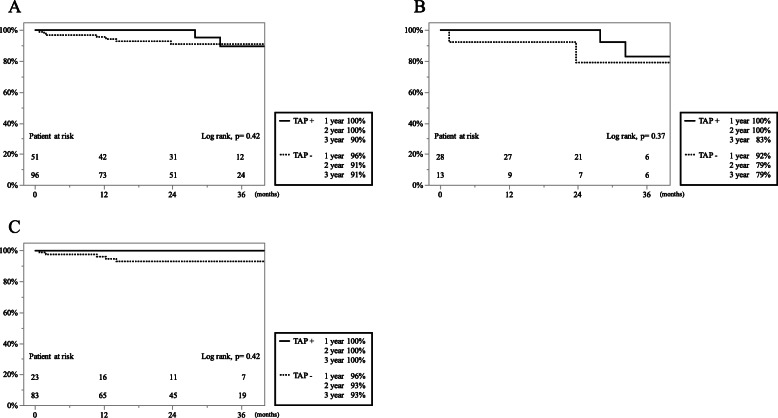
Kaplan-Meier analysis of postoperative tricuspid regurgitation (TR)-free rates (TR ≤ mild) is shown according to concomitant tricuspid annuloplasty (TAP). **a** The overall TR-free rates in patients with (solid line) and without (dotted line) concomitant TAP. **b** Patients with preoperative TR (*n* = 41). **c** Patients without preoperative TR (*n* = 106)

The Kaplan–Meier analysis of postoperative TR-free rates (TR ≤ mild) is shown in Fig. 3. The TR free rates were 100, 100, and 90% at 1, 2, and 3 years in the 51 patients with concomitant TAP and 96, 91, and 91% in the 96 patients without concomitant TAP (*p* = 0.42), respectively (Fig. 3a). In the TR group, the TR free rates were 100, 100, and 83% at 1, 2, and 3 years in patients with concomitant TAP and 92, 79, and 79% without TAP (*p* = 0.37), respectively (Fig. 3b). In contrast, in the non-TR group, the TR free rates were 100, 100, and 100% at 1, 2, and 3 years in patients with concomitant TAP and 96, 93, and 93% in patients without TAP (*p* = 0.42), respectively (Fig. 3c).

Conversely, during follow-up, sixteen (11%) patients had late RV failure (Supplement 2). The characteristics of patients with or without late RV failure are described in Table 3. Patients with dilated phase hypertrophic cardiomyopathy (dHCM) or a pacing device showed higher rates of late RV failure. Regarding echocardiography data, the preoperative dimension of left ventricle was smaller in patients with late RV failure than in patients without late RV failure. However, TR development during LVAD support (except for TR worsening after or during RV failure development) did not differ between patients with or without late RV failure. The preoperative risk factors for late RVF with *P* < 0.05 on univariate analysis were as follows: dHCM, pacing device, left ventricular end-diastolic dimension (LVDd), left ventricular end-systolic dimension, and left ventricular ejection fraction. According to multivariate analysis including these factors, the pacing device was the independent risk factor of late RVF (hazard ratio, 3.75; 95% CI: 1.11–17.8, *p* = 0.03).

**Table 3:** Comparison between patients with and without late RVF

	no late RVF	late RVF	Univariable analysis	*p* value	Multivariable analysis	*p* value
	(*n* = 131)	(*n* = 16)	HR (95% CI)		HR (95% CI)	
Etiology						
DCM, n(%)	82 (63%)	7 (44%)	0.38 (0.13–1.06)	0.06		
dHCM, n(%)	15 (11%)	7 (44%)	5.11 (1.79–14.2)	0.0032*	2.89 (0.87–9.15)	0.08
ICM, n(%)	15 (11%)	0 (0%)	–	0.07		
pacing device, n(%)	67 (52%)	13 (81%)	3.77 (1.21–16.5)	0.02*	3.75 (1.11–17.8)	0.03*
TR worsening during LVAD						
(except for TR worsening after RVF)	6 (5%)	0 (0%)	–	0.28		
Pre-LVAD UCG						
LVDd (mm)	70.4 ± 12.1	63.3 ± 15.7	0.95 (0.91–0.99)	0.01*	0.87 (0.69–1.08)	0.20
LVDs (mm)	65.0 ± 12.8	57.0 ± 17.8	0.96 (0.92–0.99)	0.01*	1.10 (0.89–1.36)	0.38
LVEF (%)	20.1 ± 8.1	25.6 ± 11.0	1.06 (1.01–1.11)	0.02*	1.03 (0.94–1.12)	0.55
RVDd (mm)	39.8 ± 10.1	41.0 ± 9.0	1.01 (0.96–1.07)	0.62		
TAPSE (mm/s)	14.5 ± 4.7	12.7 ± 4.3	0.92 (0.80–1.04)	0.19		
TRPG (mmHg)	32.0 ± 14.0	27.6 ± 13.4	0.96 (0.91–1.00)	0.07		
Pre-LVAD RHC						
PAP systolic (mmHg)	40.0 ± 13.3	39.6 ± 15.3	0.98 (0.95–1.02)	0.39		
PAP diastolic (mmHg)	19.8 ± 7.9	19.0 ± 8.9	0.97 (0.91–1.03)	0.30		
PAP mean (mmHg)	27.8 ± 9.6	27.6 ± 11.1	0.98 (0.93–1.03)	0.39		
PCWP (mmHg)	20.1 ± 8.3	19.8 ± 10.5	0.97 (0.92–1.03)	0.37		
RAP (mmHg)	7.6 ± 4.8	9.6 ± 4.8	1.07 (0.97–1.17)	0.18		
CI(L/min/m2)	2.25 ± 0.80	2.26 ± 0.52	1.09 (0.56–1.74)	0.78		
RAP/PCWP	0.39 ± 0.30	0.50 ± 0.22	2.55 (0.61–6.47)	0.17		
PAPi	2.86 (0.55–17)	2.17 (0.81–16)	0.93 (0.74–1.10)	0.47		
RVSWI (mmHg×ml/m2)	537 ± 281	533 ± 277	1.00 (1.00–1.00)	0.71		
Pre-LVAD laboratory data						
BUN (mg/dl)	19.7 ± 12.5	24.1 ± 11.4	1.02 (0.99–1.05)	0.17		
Cr (mg/dl)	1.04 ± 0.49	1.18 ± 0.47	1.50 (0.57–3.12)	0.38		
Ccr (ml/min/m2)	72.9 ± 35.3	56.7 ± 23.4	0.98 (0.96–1.00)	0.08		
T-Bil (mg/dl)	1.07 ± 0.77	1.02 ± 0.51	0.94 (0.38–1.76)	0.87		
BNP (pg/μl)	388 (30.8–4206)	561 (44–3022)	1.00 (1.00–1.00)	0.50		

*BNP* brain natriuretic peptide, *BUN* blood urea nitrogen, *Ccr* creatinine clearance, *CI* cardiac index, *Cr* serum creatinine, *DCM* dilated cardiomyopathy, *dHCM* dilated phase hypertrophic cardiomyopathy, *HR* hazard ratio, *ICM* ischemic cardiomyopathy, *LVAD* left ventricular assist device, *LVDd* left ventricular end-diastolic dimension, *LVDs* left ventricular end-systolic dimension, *LVEF* left ventricular ejection fraction, *PAP* pulmonary artery pressure, *PAPi* pulmonary artery pulsatility index, *PCWP* pulmonary capillary wedge pressure, *RAP* right atrial pressure, *RHC* right heart catheterization, *RVDd* right ventricular end diastolic dimension, *RVF* right ventricular failure, *RVSWI* right ventricular stroke work index, *TAPSE* tricuspid annular plane systolic excursion, *T-Bil* total bilirubin, *TR* tricuspid regurgitation, *TRPG* tricusid regurgitation peak gradient, *UCG* ultrasonic echocardiography

**Fig. 4:**
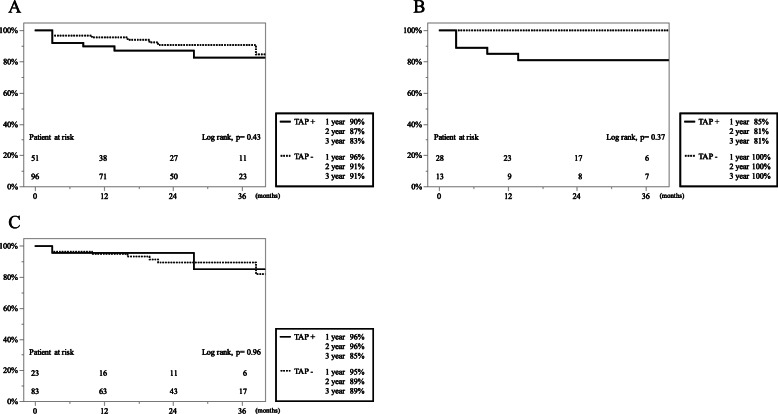
The Kaplan-Meier analysis of late right ventricular failure (RVF)-free rates is shown according to concomitant tricuspid annuloplasty (TAP). **a** The overall late RVF-free rates in patients with (solid line) and without (dotted line) concomitant TAP. **b** Patients with preoperative tricuspid regurgitation (TR) (*n =* 41). **c** Patients without preoperative TR (*n* = 106)

The Kaplan–Meier analysis of late RV failure-free rates is shown in Fig. 4. The late RV failure-free rates were 90, 87, and 83% at 1, 2, and 3 years in the 51 patients with concomitant TAP and 96, 91, and 91% in the 96 patients without concomitant TAP (*p* = 0.43), respectively (Fig. 4a). In the TR group, the late RV failure-free rates were 85, 81, and 81% at 1, 2, and 3 years in patients with concomitant TAP and 100, 100, and 100% without TAP (*p* = 0.37, Fig. 4b), respectively. By contrast, in the non-TR group, the RV failure-free rates were 96, 96, and 85% at 1, 2, and 3 years in patients with concomitant TAP and 95, 89, and 89% in patients without TAP (*p* = 0.96, Fig. 4c), respectively.

## Discussion

The primary findings of the present study were as follows: (1) patients with larger RVDd and higher serum BUN levels required unexpected TAP, even for patients without preoperative TR; (2) the occurrence of postoperative TR was consistent between patients with or without concomitant TAP; (3) the development of late RV failure independently occurred, despite postoperative TR management.

In our analysis, 9 of 92 patients without preoperative TR required unexpected TAP. The univariate analysis revealed that dilated preoperative RVDd and elevated serum BUN levels were predictors of unexpected TAP. There have been various reports that dilated tricuspid annulus and/or RV were predictors of the occurrence of postoperative TR [18]. In our patients, those who required unexpected TAP had significantly higher serum BUN/Cr levels. Patients with end-stage heart failure are often stabilized in a slightly dehydrated state owing to inotrope support and diuretics, which can be evaluated by BUN/Cr levels; preoperative TR in these dehydrated patients could be underestimated. When these patients receive LVAD implantation under CPB, unexpected TR can occur because adequate volume must be loaded to achieve sufficient LVAD flow.

In our study, TR prognosis during LVAD support was similar between patients with and without concomitant TAP, regardless of preoperative TR status. In patients without preoperative TR, the occurrence of post-LVAD TR worsening was very low. This result implies that prophylactic TAP is not necessary for LVAD implantation patients. In patients with preoperative TR, the late TR prognosis was also favorable. The reason for this similarity can be explained by the etiology of TR. Patients with heart failure often have TR, in which the etiologies include primary RV dilatation, pulmonary hypertension (PH) and device-related TR due to RV lead adhesion [19–21]. In this study, patients with preoperative TR had significantly higher PAP and CVP, as well as dilated RV. This result suggests that the main cause of preoperative TR was PH rather than dilated RV cardiomyopathy. Unloading of the LV and left atrium pressure after LVAD implantation decreases PH, which consequently improved the TR. Concomitant TAP may not be necessary for patients whose preoperative TR is estimated to be caused by PH [15, 22, 23].

However, the mechanism of postoperative TR remains unknown. In our patients, postoperative TR developed in 9 patients. In 2 of 9 patients, RVDd was severely dilated and larger than LVDd when TR grade was severe. In addition, the TRPG was relatively low (6 and 10 mmHg), and the tricuspid annulus diameter was well controlled below 40 mm owing to TAP. According to these results, the mechanism of late TR can be explained by the ventricular septal shift to the left side because of LV unloading or primary RV dilatation due to impaired RV contraction (primary RV failure).

Furthermore, the impact of postoperative TR on late RV function has not been determined [8, 15, 24]. In this study, 6 of 9 patients with postoperative TR did not have late RV failure. Conversely, late RV failure developed in 16 of 147 patients. Thirteen of these sixteen patients did not have TR during LVAD support, while the other patients had postoperative TR after late RV failure occurred. These results may suggest that the postoperative TR has little impact on late RV failure. In this study, the independent risk factor of late RV failure was a pacing device, but TR worsening during LVAD (except for TR worsening after RV failure) was not a risk factor of late RV failure. This result implies that TR induced by pacing lead has little impact on the occurrence of late RV failure. LVAD patients implanted pacing devices often have advanced myocardial damage. In such patients, damage to the RV may progress even after LVAD implantation, resulting in late RV failure. In patients with late RV failure, the mechanism of RV failure is not the increment of RV preload due to TR, but rather the impairment of RV contractility due to cardiomyopathy or the geometric impairment due to the septal shift.

Our study has several limitations. First, it was a single-center retrospective study with a relatively limited number of patients. Second, the follow-up period was short because most patients underwent heart transplantation within 4 years. Third, it included several kinds of LVADs including centrifugal and axial pumps. Device differences may have impacted LV unloading and septal shift, which can cause RV failure or TR worsening. Finally, the definition of RV failure depended on clinical symptoms. An evaluation using RHC may identify patients with potential RV failure and can provide different results.

## Conclusions

The prognosis of TR due to pulmonary hypertension was preferable regardless of concomitant TAP; however, late RV failure sometimes occurred, even in patients without TR worsening. Further evaluation of the detailed mechanism and management of late TR and RV failure are now required. It is important to realize that concomitant TAP is still necessary for patients with preoperative TR. However, it is also important to be aware that late RV failure cannot be prevented by prophylactic TAP.

## Supplementary Information

**Additional file 1.**

## Data Availability

The datasets used for the this study are available from the corresponding author on reasonable request.

## Abbreviations

BUN: Blood urea nitrogen
CPB: Cardiopulmonary bypass
Cr: Serum creatinine
dHCM: Dilated phase hypertrophic cardiomyopathy
IABP: Intra-aortic balloon pumping
LVAD: Left ventricular assist device
PAPi: Pulmonary artery pulsatility index
PCWP: Pulmonary capillary wedge pressure
RAP: Right atrium pressure
RHC: Right heart catheterization
RV: Right ventricular
RVDd: Right ventricular end-diastolic dimension
RVSWI: Right ventricular stroke work index
TAP: Tricuspid annuloplasty
TAPSE: Tricuspid annular plane systolic excursion
TR: Tricuspid regurgitation
TRPG: Tricuspid regurgitation peak gradient

## References

[1] Rose EA, Gelijns AC, Moskowitz AJ, Heitjan DF, Stevenson LW, Dembitsky W, Long JW, Ascheim DD, Tierney AR, Levitan RG, Watson JT, Ronan NS, Shapiro PA, Lazar RM, Miller LW, Gupta L, Frazier OH, Desvigne-Nickens P, Oz MC, Poirier VL, Meier P (2001). Long-term use of a left ventricular assist device for end-stage heart failure. N Engl J Med.

[2] Slaughter MS, Rogers JG, Milano CA, Russell SD, Conte JV, Feldman D, Sun B, Tatooles AJ, Delgado RM, Long JW, Wozniak TC, Ghumman W, Farrar DJ, Frazier OH (2009). Advanced heart failure treated with continuous-flow left ventricular assist device. N Engl J Med.

[3] Kormos RL, Teuteberg JJ, Pagani FD, Russell SD, John R, Miller LW, Massey T, Milano CA, Moazami N, Sundareswaran KS, Farrar DJ, HeartMate II Clinical Investigators (2010). Right ventricular failure in patients with the HeartMate II continuous-flow left ventricular assist device: incidence, risk factors, and effect on outcomes. J Thorac Cardiovasc Surg.

[4] Matthews JC, Koelling TM, Pagani FD, Aaronson KD (2008). The right ventricular failure risk score a pre-operative tool for assessing the risk of right ventricular failure in left ventricular assist device candidates. J Am Coll Cardiol.

[5] Saito S, Sakaguchi T, Miyagawa S, Nishi H, Yoshikawa Y, Fukushima S, Daimon T, Sawa Y (2012). Recovery of right heart function with temporary right ventricular assist using a centrifugal pump in patients with severe biventricular failure. J Heart Lung Transplant.

[6] Kirklin JK, Naftel DC, Kormos RL, Stevenson LW, Pagani FD, Miller MA, Baldwin JT, Young JB (2013). Fifth INTERMACS annual report: risk factor analysis from more than 6,000 mechanical circulatory support patients. J Heart Lung Transplant.

[7] Baumwol J, Macdonald PS, Keogh AM, Kotlyar E, Spratt P, Jansz P, Hayward CS (2011). Right heart failure and "failure to thrive" after left ventricular assist device: clinical predictors and outcomes. J Heart Lung Transplant.

[8] Fujita T, Kobayashi J, Hata H, Seguchi O, Murata Y, Yanase M, Nakatani T (2014). Right heart failure and benefits of adjuvant tricuspid valve repair in patients undergoing left ventricular assist device implantation. Eur J Cardiothorac Surg.

[9] Fukamachi K, McCarthy PM, Smedira NG, Vargo RL, Starling RC, Young JB (1999). Preoperative risk factors for right ventricular failure after implantable left ventricular assist device insertion. Ann Thorac Surg.

[10] Kang G, Ha R, Banerjee D (2016). Pulmonary artery pulsatility index predicts right ventricular failure after left ventricular assist device implantation. J Heart Lung Transplant.

[11] Takeda K, Takayama H, Colombo PC, Yuzefpolskaya M, Fukuhara S, Han J (2015). Incidence and clinical significance of late right heart failure during continuous-flow left ventricular assist device support. J Heart Lung Transplant.

[12] Nakanishi K, Homma S, Han J, Takayama H, Colombo PC, Yuzefpolskaya M, Garan AR, Farr MA, Kurlansky P, di Tullio MR, Naka Y, Takeda K (2018). Usefulness of tricuspid annular diameter to predict late right sided heart failure in patients with left ventricular assist device. Am J Cardiol.

[13] Rich JD, Gosev I, Patel CB, Joseph S, Katz JN, Eckman PM, Lee S, Sundareswaran K, Kilic A, Bethea B, Soleimani B, Lima B, Uriel N, Kiernan M, Evolving Mechanical Support Research Group (EMERG) Investigators (2017). The incidence, risk factors, and outcomes associated with late right-sided heart failure in patients supported with an axial-flow left ventricular assist device. J Heart Lung Transplant.

[14] Veen KM, Muslem R, Soliman OI, Caliskan K, Kolff MEA, Dousma D, Manintveld OC, Birim O, Bogers AJJC, Takkenberg JJM (2018). Left ventricular assist device implantation with and without concomitant tricuspid valve surgery: a systematic review and meta-analysis. Eur J Cardiothorac Surg.

[15] Piacentino V, Ganapathi AM, Stafford-Smith M, Hsieh MK, Patel CB, Simeone AA (2012). Utility of concomitant tricuspid valve procedures for patients undergoing implantation of a continuous-flow left ventricular device. J Thorac Cardiovasc Surg.

[16] Hoeper MM, Bogaard HJ, Condliffe R, Frantz R, Khanna D, Kurzyna M, Langleben D, Manes A, Satoh T, Torres F, Wilkins MR, Badesch DB (2013). Definitions and diagnosis of pulmonary hypertension. J Am Coll Cardiol.

[17] Nishimura RA, Otto CM, Bonow RO, Carabello BA, Erwin JP, Guyton RA (2014). 2014 AHA/ACC guideline for the Management of Patients with Valvular heart disease: a report of the American College of Cardiology/American Heart Association task force on practice guidelines. Circulation..

[18] Nakanishi K, Homma S, Han J, Takayama H, Colombo PC, Yuzefpolskaya M (2018). Prevalence, predictors, and prognostic value of residual tricuspid regurgitation in patients with left ventricular assist device. J Am Heart Assoc.

[19] Abu Sham'a R, Buber J, Grupper A, Nof E, Kuperstein R, Luria D, Feinberg MS, Eldar M, Glikson M (2013). Effects of tricuspid valve regurgitation on clinical and echocardiographic outcome in patients with cardiac resynchronization therapy. Europace..

[20] Dreyfus GD, Corbi PJ, Chan KM, Bahrami T (2005). Secondary tricuspid regurgitation or dilatation: which should be the criteria for surgical repair?. Ann Thorac Surg.

[21] Ring L, Rana BS, Kydd A, Boyd J, Parker K, Rusk RA (2012). Dynamics of the tricuspid valve annulus in normal and dilated right hearts: a three-dimensional transoesophageal echocardiography study. Eur Heart J Cardiovasc Imaging.

[22] Lee S, Kamdar F, Madlon-Kay R, Boyle A, Colvin-Adams M, Pritzker M, John R (2010). Effects of the HeartMate II continuous-flow left ventricular assist device on right ventricular function. J Heart Lung Transplant.

[23] Morgan JA, Paone G, Nemeh HW, Murthy R, Williams CT, Lanfear DE, Tita C, Brewer RJ (2013). Impact of continuous-flow left ventricular assist device support on right ventricular function. J Heart Lung Transplant.

[24] Maltais S, Topilsky Y, Tchantchaleishvili V, McKellar SH, Durham LA, Joyce LD (2012). Surgical treatment of tricuspid valve insufficiency promotes early reverse remodeling in patients with axial-flow left ventricular assist devices. J Thorac Cardiovasc Surg.

