# Supplementary material for: Impact of tricuspid regurgitation on late right ventricular failure in left ventricular assist device patients ~can prophylactic tricuspid annuloplasty prevent late right ventricular failure? ~
Source: J Cardiothorac Surg. 2021 Apr 20;16:99. doi: 10.1186/s13019-021-01492-0 (PMC8056678; doi:10.1186/s13019-021-01492-0)
Supplement: Supplementary file 1 — Additional file 1. [file 13019_2021_1492_MOESM1_ESM.docx]

Supplement 1. Details of patients who had postoperative tricuspid regurgitation.

| Age, sex | Etiology | Device | pre LVDd/Ds (mm) | pre RVDd (mm) | pre TR | TAP | TR ≥ moderate period  (months) | post LVDd/Ds (mm) | post RVDd (mm) | TRPG (mmHg) | TAD (mm) | late RVF |  |
| --- | --- | --- | --- | --- | --- | --- | --- | --- | --- | --- | --- | --- | --- |
| 55 M | DCM | HeartMate II | 60/54 | 46 | 4 | - | 1.6 | 45/37 | 58 | 18 | 50 | - |  |
| 51 M | ICM | EVAHEART | 77/76 | 44 | 4 | - | 23.7 | 69/63 | 32 | 19 | 55.7 | - |  |
| 40 F | ARVC | DuraHeart | 47/40 | 46 | 3 | + | 32.3 | 33/28 | 59 | 6 | 36.7 | + |  |
| 29 M | DCM | HeartMate II | 68/63 | 59 | 3 | + | 27.9 | 58/58 | 72 | 10 | 38.6 | + |  |
|  |  |  |  |  |  |  |  |  |  |  |  |  |  |
| 21 F | dHCM | DuraHeart | 69/64 | 27 | 2 | - | 14.2 | 48/47 | 44 | 13 | 30.3 | + |  |
| 27 M | drug | HeartWare | 64/58 | 29 | 2 | - | 0.8 | 58/56 | 34 | 14 | 26.3 | - |  |
| 63 M | ICM | HeartMate II | 79/77 | 47 | 2 | - | 10.8 | 71/71 | 52 | 13 | 50.6 | - |  |
| 39 M | DCM | Jarvik 2000 | 75/68 | n/a | 1 | - | 12.4 | 72/64 | 46 | 18 | n/a | - |  |
| 37 F | DCM | DuraHeart | 81/70 | 17 | 0 | - | 1.9 | 41/35 | 50 | 12 | 38.6 | - |  |
| *ARVC: arrhythmogenic right ventricular cardiomyopathy, DCM: dilated cardiomyopathy, dHCM: dilated phase hypertrophic cardiomyopathy, F: female, ICM: ischemic cardiomyopathy, LVDd: left ventricular end-diastolic dimension, LVDs: left ventricular end-systolic dimension, M: male, n/a: not assessed, RVDd: right ventricular end-diastolic dimension, RVF: right ventricular failure, TAD: tricuspid annulus diameter, TAP: tricuspid annuloplasty, TR: tricuspid regurgitation, TRPG: tricuspid regurgitation pressure*  TR ≥ 3 period: The period until TR ≥ moderate, post LVDd/Ds: LVDd/Ds at TR ≥ moderate, post RVDd: RVDd at TR ≥ moderate, TRPG: TRPG at TR ≥ moderate, TAD: TAD at TR ≥ moderate | | | | | | | | | | | | | |

Supplement 2. Details of patients who had late right ventricular failure.

| Age,sex | Etiology | Device | pre LVDd/Ds (mm) | pre RVDd (mm) | pre PAPi | pre TR | TAP | 6-months LVDd/Ds (mm) | 6-months RVDd (mm) | 6-months TAD  (mm) | TR ≥ moderate during LVAD | |
| --- | --- | --- | --- | --- | --- | --- | --- | --- | --- | --- | --- | --- |
| 35 F | RCM | EVAHEART | 48/36 | 46 | 3.44 | 4 | + | 36/23 | 52 | 32.1 | - | |
| 29 M | DCM | HeartMate II | 68/63 | 59 | 5.00 | 3 | + | 57/55 | 72 | 33.4 | + | |
| 40 F | ARVC | DuraHeart | 47/40 | 46 | 2.00 | 3 | + | 28/24 | 50 | 38.1 | + | |
| 55 M | DCM | EVAHEART | 52/47 | 41 | 3.33 | 3 | + | 47/38 | 47 | 34.8 | - | |
| 57 M | DCM | EVAHEART | 61/59 | 50 | 2.86 | 3 | + | 59/57 | 38 | 38.3 | - | |
| 57 M | DCM | DuraHeart | 68/66 | 37 | 4.20 | 3 | - | 30/23 | 30 | 30.9 | - | |
|  |  |  |  |  |  |  |  |  |  |  |  | |
| 62 M | dHCM | HeartMate II | 88/86 | 48 | 3.40 | 2 | + | 65/60 | 57 | 32.2 | - | |
| 65 F | dHCM | HeartMate II | 48/43 | 27 | 1.86 | 2 | + | 35/27 | 27 | 28.6 | - | |
| 21 F | dHCM | DuraHeart | 69/64 | 27 | 1.64 | 2 | - | 35/32 | 44 | 28.4 | + | |
| 24 M | dHCM | DuraHeart | 45/30 | 38 | 1.18 | 2 | - | 45/32 | n/a | n/a | - | |
| 42 M | dHCM | EVAHEART | 54/43 | 38 | 1.25 | 2 | - | 48/36 | 28 | 36.2 | - | |
| 44 F | dHCM | Jarvik2000 | 48/43 | 28 | 0.81 | 2 | - | 25/21 | 26 | 35.1 | - | |
| 44 F | DCM | HeartMate II | 91/86 | 45 | 1.40 | 2 | - | 80/78 | 35 | 42.4 | - | |
| 52 M | DCM | HeartMate II | 68/62 | 35 | 2.33 | 2 | - | 61/56 | 36 | 41.3 | - | |
| 40 M | dHCM | HeartWare | 69/60 | 48 | 1.93 | 2 | - | 49/43 | 45 | 37.8 | - | |
| 47 M | DCM | HeartWare | 89/84 | 41 | 16.0 | 2 | - | 78/74 | 27 | 37.3 | - | |
| *ARVC: arrhythmogenic right ventricular cardiomyopathy, DCM: dilated cardiomyopathy, dHCM: dilated phase hypertrophic cardiomyopathy, F: female, LVAD: left ventricular assist device, LVDd: left ventricular end-diastolic dimension, LVDs: left ventricular end-systolic dimension, M: male, n/a: not assessed, PAPi: pulmonary artery pulsatility index, RCM: restrictive cardiomyopathy, RVDd: right ventricular end-diastolic dimension, RVF: right ventricular failure, TAD: tricuspid annulus diameter, TAP: tricuspid annuloplasty, TR: tricuspid regurgitation*  6-months LVDd/Ds: LVDd/Ds 6 months after LVAD implantation, 6-months RVDd: RVDd 6 months after LVAD implantation, 6-months TAD: TAD 6 months after LVAD implantation | | | | | | | | | | | |  |
